# Nutritional Modulation of Impaired Blood-Brain Barrier Integrity and Function in Major Depression

**DOI:** 10.3390/ijms26146917

**Published:** 2025-07-18

**Authors:** Miroslav Adzic, Iva Lukic, Milos Mitic, Ester Francija Zerajic, Emilija Glavonic, Milan Jovanovic, Sanja Ivkovic

**Affiliations:** 1 Department of Molecular Biology and Endocrinology, “Vinča” Institute of Nuclear Sciences-National Institute of the Republic of Serbia, University of Belgrade, 11000 Belgrade, Serbia; iva.lukic@vin.bg.ac.rs (I.L.); milos.mitic@vin.bg.ac.rs (M.M.); francijaester89@gmail.com (E.F.Z.); milan.jov94@gmail.com (M.J.); sivkovic@vin.bg.ac.rs (S.I.); 2 Department of Pharmacy, Singidunum University, 11000 Belgrade, Serbia

**Keywords:** major depressive disorder, blood-brain barrier, nutrition, neuroinflammation, dietary interventions

## Abstract

Major Depressive Disorder (MDD) is increasingly linked to disruptions in blood-brain barrier (BBB) integrity, contributing to neuroinflammation and impaired brain homeostasis. While traditional antidepressant therapies often fail to achieve full remission, growing evidence suggests that specific dietary compounds may offer novel avenues for restoring BBB function and improving mental health outcomes. This review explores the potential of selected nutrients—omega-3 fatty acids, vitamin D, sulforaphane, fucoidan, and urolithins—to modulate BBB integrity through anti-inflammatory, antioxidant, and transporter-regulatory mechanisms. These compounds act by reinforcing tight junctions, reducing matrix metalloproteinase activity, and modulating efflux transporters such as P-glycoprotein. Although current evidence is largely preclinical, the mechanistic insights provided in this review support the rationale for integrating nutritional strategies into the management of MDD. Future clinical studies are needed to validate these findings and develop biomarker-driven approaches for targeting the BBB in nutritional interventions for psychiatric disorders.

## 1. Introduction

Major depressive disorder (MDD) is a critical public health condition characterized by sustained feelings of sadness, loss of interest in life, persistent fatigue, and disturbed cognitive function [1]. MDD affects more than 300 million individuals worldwide, making it a major contributor to the global disease burden [2]. Despite several decades of research into the pharmacological treatment of depression, approximately 50% of patients with MDD fail to achieve full remission with current antidepressant (AD) treatments [3] due to the complex and multifaceted underlying pathophysiology of MDD. Namely, various hypotheses, including dysfunction of the hypothalamic-pituitary-adrenal (HPA) axis, monoaminergic dysregulation, inflammatory processes, and genetic and epigenetic factors, have been proposed to explain the etiology of MDD [4,5].

In addition to these mechanisms, increasing attention is being paid to the role of the BBB, not only as a passive physical filter but also as a dynamic interface regulating neuroimmune interactions, brain metabolism, and central access to therapeutic agents [6,7,8]. Recently, MDD has been increasingly associated with disruptions in the BBB, which are believed to disturb brain homeostasis, leading to adverse health outcomes [6]. Moreover, several studies [7,8] reported a bidirectional relationship between BBB integrity and neuroinflammation in MDD. Neuroinflammation can lead to BBB disruption, while compromised BBB integrity can exacerbate neuroinflammation by allowing peripheral inflammatory factors to access the brain. Additionally, MDD, BBB dysfunction is also implicated in several neurological disorders, including Alzheimer’s disease, Parkinson’s disease, Huntington’s disease, amyotrophic lateral sclerosis, and chronic traumatic encephalopathy, suggesting shared pathological mechanisms rooted in vascular and inflammatory dysregulation [9].

Interestingly, while the BBB is increasingly recognized as a contributor to the pathophysiology of MDD, its role in treatment response remains underexplored [10,11]. Given that BBB dysfunction alters brain homeostasis and facilitates neuroinflammation, it is plausible that such alterations may also influence the efficacy of antidepressants, particularly in treatment-resistant cases, by affecting drug delivery or local pharmacokinetics at the neurovascular interface. Although direct clinical evidence remains limited, this potential mechanism warrants further investigation, particularly in light of growing interest in non-pharmacological strategies to restore BBB function.

One such strategy involves dietary modulation. The involvement of dietary factors in BBB dysfunction associated with MDD remains inadequately explained. Nutritional factors such as omega-3 fatty acids, antioxidants, and several other micronutrients have been considered important modulators of the permeability of the BBB and neuroinflammation [12]. Therefore, the addition of these supplements may exert protective effects on the integrity of the BBB, prevent neurovascular dysfunction, and thus contribute to better mental outcomes in patients with depression. Emerging research in nutritional psychiatry increasingly emphasizes the links between nutrients, dietary patterns, and the risk of depression [12]. However, while the connection between nutrition and depression risk is gaining attention, studies specifically exploring the impact of food on the BBB in the context of psychiatric disorders remain scarce.

It is essential to note that, beyond their direct influence on the BBB, several nutritional interventions have demonstrated wider-ranging benefits in supporting and enhancing mental health. Several studies have shown that some nutritional interventions are quite important in the preservation and enhancement of mental health, especially through affecting mechanisms like neuroinflammation, oxidative stress, and neurotransmitter imbalance that are involved in MDD [13]. Foods rich in antioxidants, vitamins, minerals, and bioactive substances promote neurobiological mechanisms underlying psychiatric disorders [14]. In particular, dietary interventions, such as increased intake of polyphenols, omega-3 fatty acids, B vitamins, and probiotics, have also proven effective in treating depressive symptoms by targeting these mechanisms. For example, polyphenols possess anti-inflammatory and antioxidant abilities that neutralize neuroinflammation and oxidative stress within the brain [15]. Omega-3 fatty acids are implicated in fluidity within neuronal membranes and modulate inflammatory signaling [16], while B vitamins serve as crucial cofactors in the biosynthesis of neurotransmitters [17]. Furthermore, the gut-brain axis, which is modulated by probiotic supplementation, is now recognized as a critical factor in mental health, and it affects mood regulation through immune and neuroendocrine mechanisms [18]. Taken together, the findings suggest that individually tailored nutritional treatments may offer effective adjunct therapies in MDD, improving the response to treatment and complementing existing therapy.

Given the accessibility, safety, and low cost of nutritional approaches, identifying specific dietary compounds that support BBB integrity could offer practical adjunctive strategies in MDD management. Therefore, this review places dietary interventions as complementary approaches in the treatment of MDD, exploring how specific nutrients, according to current evidence, might influence the course of BBB functioning and neuroinflammation. A better understanding of the complex interaction between diet and BBB health might lead to new solutions for improving the quality of life of people living with depression.

### 1.1. The Blood-Brain Barrier Composition

The BBB plays a vital role within the central nervous system (CNS), serving as a highly selective filter that controls the exchange of molecules between the brain and the bloodstream [8]. The structure of the BBB is predominantly made up of endothelial cells that envelop the cerebral microvasculature. These cells are closely connected by complex junctional proteins, including tight junctions (TJs) and adherens junctions, which significantly restrict paracellular permeability [8]. The TJs are mainly formed by claudins, occludins, and junctional adhesion molecules (JAMs), which seal the gaps between endothelial cells, preventing the free passage of solutes [9]. Claudin-5 and occludin are highly expressed in CNS endothelial cells, while their respective depletions have been associated with heightened permeability of the BBB.

**Figure 1:**
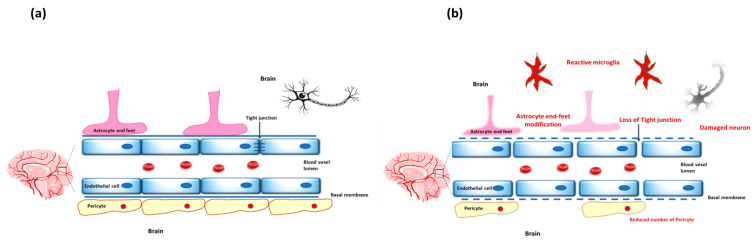
The Blood-Brain Barrier: Differences Between Healthy (**a**) and MDD Conditions (**b**): Panel (**a**) shows the intact BBB in healthy conditions, with well-preserved tight junction proteins, normal transporter activity, and astrocyte support, maintaining brain homeostasis. Panel (**b**) illustrates BBB disruption in MDD, featuring loss of tight junction integrity, increased permeability, neuroinflammation, oxidative stress, and impaired transporter function. These changes contribute to barrier breakdown and promote depressive pathology.

Beyond endothelial cells, pericytes and astrocytic end-feet are key components essential for the BBBs’ structural and functional integrity, forming a neurovascular unit (NVU) [19]. Pericytes are embedded within the basement membrane and play a role in BBB stability, regulating endothelial cell proliferation and survival, and controlling blood flow within the brain [20]. They are particularly abundant in the CNS [21] and share a basement membrane with endothelial cells [22]. With their end-feet surrounding the capillaries, astrocytes contribute biochemical support to endothelial cells and play a vital role in maintaining the BBB’s selective permeability [23], Figure 1a.

At the BBB, specialized membrane-bound proteins known as transporters, particularly abundant in brain endothelial cells, are essential for maintaining selective permeability [24]. These transporters tightly regulate the entry of vital nutrients and other necessary circulating molecules while actively preventing the passage of potentially harmful substances [25]. Although highly concentrated in the brain, these proteins are also expressed in other barrier-forming organs such as the placenta and kidneys, underscoring their critical function in preserving systemic homeostasis [25]. BBB transporters are mainly grouped into two major superfamilies: the ATP-binding cassette (ABC) transporters [26] and the solute carrier (SLC) transporters [27]. Together, these families coordinate the controlled exchange of a wide range of substances between the bloodstream and the brain, playing a central role in protecting and supporting neural function. In particular, ABC transporters act like cellular pumps, using energy from ATP hydrolysis to actively expel various molecules from brain endothelial cells back into the bloodstream. Their primary role is protective, preventing potentially harmful substances from entering the brain [28]. Some of the most studied ABC transporters at the BBB include [29]: P-glycoprotein (ABCB1/P-gp), which restricts drug entry into the brain; Breast Cancer Resistant Protein (ABCG2/BCRP), involved in drug and toxin efflux; ABCG4, specific to brain endothelial cells (BECs) and linked to cholesterol transport; Multidrug Resistance Proteins 1 and 2 (ABCC1/MRP1 and ABCC2/MRP2), mediating efflux of various substrates; and ABCA1 and ABCA7, which regulate cholesterol and lipid homeostasis.

In contrast, SLC transporters generally mediate the movement of small molecules into the brain. They primarily function through passive transport, moving substances along their concentration gradient, from areas of higher to lower concentration. Unlike the highly selective ABC transporters, SLC transporters are more permissive and can carry a broader range of small molecules into the brain [27]. Although there are more than 300 identified SLC transporters, only a subset has been recognized for their clinical significance in the brain, primarily due to their crucial roles in nutrient transport, neurotransmitter regulation, and the movement of therapeutic drugs across the blood–brain barrier. Key examples include [30]: SLC2A1/GLUT1, which transports glucose essential for brain energy; SLC59A1/Mfsd2a, critical for DHA (an omega-3 fatty acid) transport across the BBB; SLCO1A2/OATP1A2, involved in uptake of organic anions, hormones, and drugs; SLC22A8/OAT3, contributing to clearance of metabolites and drugs; SLC7A5/LAT1, transporting large neutral amino acids to the brain; and SLC22A3/OCT3, mediating uptake of neurotransmitters and drugs, affecting physiological and pharmacological processes.

Additionally, transporters, other molecules contribute to the maintenance of BBB composition and function. Namely, Aquaporin-4 (AQP4) is a water channel protein found in the end-feet of astrocytes—cells that surround blood vessels in the brain. It helps regulate the movement of water between blood vessels and brain tissue, which is essential for maintaining a healthy brain environment and clearing out waste. AQP4 plays a key role in the glymphatic system, a brain-wide cleaning process that relies on water flow to move cerebrospinal fluid and remove metabolic waste [31].

### 1.2. Function of the Blood-Brain Barrier

The BBB is a highly selective and dynamic structure that plays a fundamental role in preserving the homeostasis of the CNS. Its primary function is to protect the brain from potentially harmful fluctuations in the systemic circulation by tightly regulating the exchange of substances between the blood and the brain parenchyma. This is achieved through a combination of physical, metabolic, and transport barriers formed by specialized endothelial cells connected by TJs, supported by pericytes, astrocytic end-feet, and the extracellular matrix [32].

A core protective role of the BBB lies in its capacity to restrict the entry of neurotoxic substances, including pathogens, circulating toxins, and even certain endogenous molecules that may disrupt neuronal signaling [7]. At the same time, it allows highly selective transport of essential nutrients such as glucose and amino acids, necessary for neuronal metabolism and function [32]. By maintaining this selective permeability, the BBB ensures a stable microenvironment critical for proper synaptic transmission and neural network integrity. Moreover, the BBB plays a crucial role in integrating peripheral signals with brain functions, influencing processes such as feeding behavior and cognition by regulating the brain’s exposure to circulating molecules [33].

Beyond its role in nutrient transport and toxin exclusion, the BBB serves as a chemical barrier by maintaining a distinct separation between neurotransmitters in the CNS and those present in peripheral circulation [34]. Circulating neurotransmitters such as dopamine or glutamate, if allowed to enter the brain freely, could cause aberrant signaling and neurotoxicity [35]. Thus, the BBB safeguards neural function by preventing peripheral neurotransmitter fluctuations from influencing CNS activity.

Another critical, yet often underappreciated, function of the BBB is its role in immunological surveillance and exclusion. Traditionally, the brain was considered immune-privileged due to the limited entry of immune cells. However, during systemic or CNS-specific inflammation, the integrity of endothelial TJs can be compromised, allowing immune cells to infiltrate the brain. This process occurs via two principal mechanisms: paracellular migration, where immune cells pass between endothelial cells due to TJ disruption, and transcellular migration, which involves passage through the endothelial cells themselves [36,37]. Under inflammatory conditions, endothelial cells upregulate adhesion molecules such as VCAM-1 and ICAM-1, which interact with VLA-4 and LFA-1 on CD4+ T cells to facilitate their attachment and migration across the BBB [38]. Additional molecules, such as melanoma cell adhesion molecule (MCAM), have been identified as key mediators of Th17 cell transmigration through the transcellular pathway [39]. In contrast, single-chain type-1 glycoprotein (CD99) plays a central role in regulating paracellular migration [40]. Its inhibition has been shown to reduce leukocyte infiltration and ameliorate disease symptoms in models of neuroinflammation, such as experimental autoimmune encephalomyelitis (EAE).

Collectively, these findings underscore the BBB’s dual role as both a physical and immunological gatekeeper. While it restricts harmful agents and maintains CNS homeostasis under physiological conditions, it also dynamically responds to pathological stimuli such as inflammation, with alterations in barrier integrity, transporter function, and immune cell trafficking.

### 1.3. The Blood-Brain Barrier Alterations in MDD

Increasing evidence suggests that the integrity of the BBB is compromised in major depression. Specifically, in patients with MDD, endothelial function is frequently impaired, as evidenced by a consistently lower relative uptake ratio (RUR) of blood flow in the brachial artery after a hyperemic challenge [41]. Furthermore, the serum of MDD patients induces greater apoptosis of endothelial cells in vitro compared to the serum of non-depressed individuals [42]. Also, stress-vulnerable mice have been shown to develop decreased expression of the endothelial tight junction molecule claudin-5 (Cldn5) and abnormal blood vessel morphology in the nucleus accumbens (NAc) [43]. This supports the strong bidirectional link between MDD and the progression of vascular endothelial pathologies [44]. Notably, MDD is highly comorbid with cardiovascular diseases, with a greater prevalence of MDD in patients with cardiovascular conditions and an elevated risk of cardiovascular issues in those with MDD [45]. Further evidence includes the reduced expression of TJ protein, claudin-5, in the hippocampus of MDD patients [6]. Moreover, the expression levels of claudin-5, claudin-12, and ZO-1 are positively linked to the age of onset and the duration of the depressive episode [6]. Imaging studies reveal that enhanced BBB leakage in bipolar patients is associated with more severe depression [46]. The extensive BBB leakage observed in this study was linked to increased severity of depression, anxiety, and socio-occupational dysfunction.

Altered expression of transporters has been linked not only to the diagnosis of MDD but also to variability in treatment response. In MDD patients, profound loss and reorganization of astrocytes lead to significant structural changes in the BBB, including approximately a 50% reduction in vascular coverage by astrocytic end-feet—particularly those immunoreactive to AQP4 in the orbitofrontal cortex—pointing to disruption of the neurovascular unit [47]. These alterations may contribute to dysregulation of cerebral blood flow, impaired glucose transport and metabolism, monoaminergic imbalances, disrupted glutamate turnover, and deficits in synaptic plasticity. Furthermore, chronic stress, a major contributor to depression, has been shown to increase AQP4 expression in the hippocampus, whereas its downregulation has been associated with reduced depressive-like behavior, potentially through modulation of NMDA receptor activity. Genetic variants in *AQP4* are also linked to increased risk for depression, implicating this astrocytic water channel in the disorder’s pathophysiology [48]. Interestingly, chronic stress may also reduce AQP4 levels, impairing glymphatic clearance and promoting β-amyloid (Aβ) accumulation, suggesting a mechanistic link between depression and neurodegenerative processes like Alzheimer’s disease [49]. Additionally, the presence of AQP4 autoantibodies in some individuals with treatment-resistant depression suggests a potential autoimmune component [50]. AQP4 also appears critical for neurogenesis and behavioral regulation, as mice lacking AQP4 display exaggerated depressive-like behaviors and diminished responsiveness to antidepressants [51]. Overall, these findings underscore the central role of AQP4 in maintaining astrocyte integrity, BBB function, and antidepressant efficacy.

One important factor influencing antidepressant effectiveness is the activity of transport proteins at the BBB that regulate drug penetration into the brain. The *ABCB1* gene, which encodes the efflux transporter P-glycoprotein (P-gp), plays a key role in controlling how much of an antidepressant reaches the brain. Genetic variations in *ABCB1* can significantly affect treatment outcomes. For example, polymorphisms like rs2235040, rs9282564, and G2677T [52] have been linked to better remission rates and faster response in patients treated with P-gp substrate antidepressants. Some variants, such as G2677T, are even associated with a higher risk of suicide attempts, highlighting their potential relevance in identifying vulnerable patients [53]. While certain ABCB1 variants do not necessarily increase the risk of developing depression, they can still influence treatment response, depending on genetic and ethnic background. Variants like 1236T, 3435T, and 2677T/A are associated with more severe initial symptoms and reduced antidepressant efficacy [54]. Importantly, these effects seem specific to drug transport and do not extend to broader endocrine systems like cortisol regulation [55]. Furthermore, both preclinical and clinical studies have indicated alterations in P-glycoprotein in the BBB upon antidepressant treatments. In particular, a clinical PET study using [^11^C] verapamil as a marker of P-gp function provided evidence of increased P-gp activity in long-term treated patients [56], which can explain treatment resistance in MDD, as P-gp acts as an efflux pump, limiting antidepressant accumulation in the CNS. An animal study documented that P-glycoprotein function at the BBB is inhibited by chronic stress and increased by chronic venlafaxine administration, highlighting the significant role of the BBB in stress-related disorders and antidepressant treatment [57]. Specifically, while chronic stress weakens the protective role of the BBB by reducing P-gp function, venlafaxine treatment restores BBB integrity by enhancing P-gp function, thereby limiting the brain’s exposure to harmful neurotoxic and inflammatory factors. Indeed, animal models of neuroinflammation have shown that pro-inflammatory cytokines downregulate P-gp function, consequently inhibiting BBB function [58]. In summary, *ABCB1* genetic variations may help predict antidepressant response and guide personalized treatment strategies, though more research is needed before routine clinical use.

Dysregulation of P-glycoprotein not only affects drug transport but is also associated with compromised BBB integrity, resulting in hyperpermeability observed in MDD. Namely, hyperpermeability of the BBB in MDD is characterized by changes in the cerebrospinal fluid (CSF)-blood ratio of various molecules, due to the decreased expression of the multidrug efflux transporter P-Glycoprotein on endothelial cells [59]. For example, the CSF/serum ratio of albumin is higher in MDD patients [60], suggesting a greater influx of albumin into the brain, which could contribute to brain tissue damage. In contrast, endothelial P-glycoprotein expression is reduced in MDD patients [56], leading to decreased expulsion of molecules from the brain and potential brain damage. In addition, the BBB hyperpermeability found in stressed rats and patients with MDD may result from an increase in pericyte number in the hippocampus, potentially driven by stress-induced proliferation mediated by hormones and cytokines [61].

Biomarkers indicating blood-brain barrier disruption and endothelial dysfunction have been increasingly observed in patients with MDD. Indeed, increased levels of S100B, a calcium-binding protein produced by glial cells, in the blood of MDD patients further point to augmented BBB leakage [62]. Also, several plasma markers of endothelial dysfunction, such as soluble ICAM-1, soluble VCAM-1, soluble E-selectin, and von Willebrand factor (vWF), are increased in patients with depression [63,64,65,66].

It was once believed that diseases cause widespread BBB disruption; however, recent evidence suggests that alterations may be localized, as in the case of depression. Namely, it has been shown that volume transfer constant (Ktrans) values, indicative of BBB permeability, are elevated in specific brain regions such as the olfactory region, caudate, and thalamus in patients with MDD compared to healthy controls. Moreover, the degree of BBB dysfunction correlates with symptom severity, as evidenced by the positive associations between Ktrans values in the hippocampus, thalamus, and white matter with HAMD and HAMA scores [67]. It has also been observed that Ktrans values in the orbital lobe, anterior cingulate gyrus, putamen, and thalamus were reduced in treated patients relative to untreated individuals. These findings not only support the notion of localized BBB disruption in MDD but also emphasize the potential of conventional antidepressant treatments to normalize BBB parameters.

Additionally, clinical findings, substantial preclinical evidence strongly suggests a link between BBB disruption and depression-like behavior, highlighting a direct connection between neurovascular health and stress vulnerability in male mice [43]. Social stress has been shown to induce regional neurovascular alterations in both female mice and women with MDD [6]. Specifically, the downregulation of the TJ protein claudin-5 in the prefrontal cortex promotes anxiety-like and depression-like behaviors, including social avoidance, in females [6]. This loss of claudin-5 expression was also confirmed in postmortem brain samples from women with MDD, underscoring its relevance to human depression. Claudin-5, which is highly expressed in CNS endothelial cells, is crucial for maintaining BBB integrity, and its depletion in mice significantly increases BBB permeability [68,69].

Overall, alterations in BBB integrity are one of the critical features of MDD, with reduced TJ proteins like claudin-5, increased permeability, and transporter dysfunction linking BBB disruption to depressive symptoms. These findings highlight the impact of neurovascular health on MDD, suggesting potential therapeutic targets.

### 1.4. The Role of Nutrients in Blood-Brain Barrier Health

The BBB is essential for brain protection, and its integrity can be affected by a range of bioactive nutrients. Compounds such as fucoidan, sulforaphane, vitamin D, urolithins, and polyunsaturated fatty acids (PUFAs) have been shown to support BBB function. These nutrients act through antioxidant, anti-inflammatory, and neuroprotective mechanisms, offering potential therapeutic benefits for preserving BBB integrity and mitigating damage in CNS disorders. In this review, we elaborated on the nutrients that directly influence the molecular and functional processes of the BBB. These compounds were selected based on their dual ability to modulate neuroinflammation and oxidative stress while also exerting direct effects on BBB structure and function, such as the regulation of tight junction proteins, preservation of endothelial cell integrity, and modulation of transporter expression. Although other bioactive agents like curcumin, resveratrol, or magnesium also exhibit neuroprotective effects, their direct actions on BBB components in the context of depression are less consistently characterized. Therefore, our focus was narrowed to compounds with more robust and specific evidence of BBB modulation relevant to MDD pathophysiology.

### 1.5. The Effects of Fucoidan on the Blood-Brain Barrier

Fucoidan, a sulfated polysaccharide found in brown algae, demonstrates potential in protecting and enhancing BBB function. It protects brain microvascular endothelial cells (BMECs) against diesel exhaust particle (DEP)-induced disruption by suppressing oxidative stress and reducing permeability [70]. In particular, while treatment of BMECs with DEP induced cellular damage and decreased TJ proteins, such as ZO-1, resulting in perforations in the cell monolayers, pretreatment with fucoidan restores ZO-1 expression and preserves barrier function.

Furthermore, fucoidan ameliorates LPS-induced neuronal damage and cognitive impairment in mice by reducing BBB permeability, inhibiting neuroinflammation, and promoting neurogenesis [71]. Although primarily studied in the gut, fucoidan’s upregulation of claudin-1 expression supports its general barrier-stabilizing effects [72].

Beyond its direct effects on the BBB, fucoidan is well-documented for its antidepressant-like effects in animal models. Chronic administration reduces depressive behaviors via hippocampal anti-inflammatory effects and enhanced BDNF-mediated synaptic plasticity [73]. Fucoidan also mitigates depressive symptoms in models of ulcerative colitis and alcohol withdrawal by modulating the gut-brain axis and restoring microbiota balance [74,75]. Furthermore, fucoidan alleviates chronic colitis and behavioral deficits by reshaping gut microbiota, enhancing colonic barrier integrity, and decreasing neuroinflammation [76]. Although these studies did not directly investigate the effect of fucoidan on the BBB, they suggest that the presence of prolonged and severe systemic inflammation may affect BBB permeability. This heightened permeability could allow more solutes, lymphocytes, and innate immune cells to cross into the brain, potentially causing endothelial cell damage, neuronal dysfunction, and clinical symptoms of depression.

**Figure 2:**
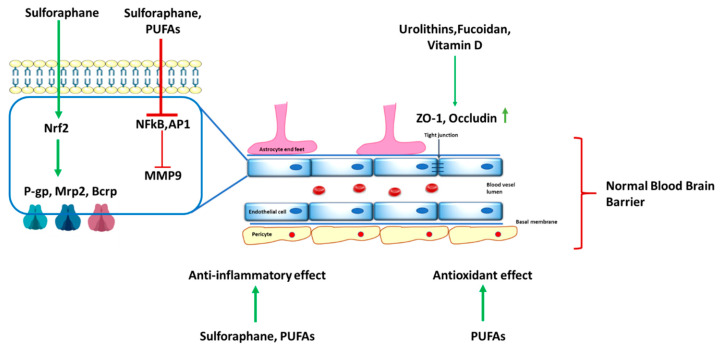
The impact of the nutrients on the blood-brain barrier: Sulforaphane: Activates the Nrf2 pathway, leading to an upregulation of efflux transporters (P-gp, Mrp2, Bcrp) that protect the BBB; exerts an anti-inflammatory effect by inhibiting NF-κB and AP1, resulting in the suppression of MMP9, which is implicated in BBB disruption. Polyunsaturated Fatty Acids (PUFAs): Contribute to the inhibition of NF-κB and AP1, helping to maintain BBB integrity; Urolithins, Fucoidan, and Vitamin D: enhance the expression of tight junction proteins (ZO-1, occludin), which are crucial for maintaining BBB structure and function. Euterpe Genus (Açaí) Supplementation: Provides an antioxidant effect, protecting the BBB from oxidative stress. P-gp—P-glycoprotein; Mrp2—multidrug resistance-associated protein-2; and Bcrp—breast cancer resistance protein.

In conclusion, fucoidan exerts both direct and indirect protective effects on the BBB, enhancing TJ protein expression, reducing oxidative and inflammatory damage, and modulating neuroimmune communication (Figure 2 and Table 1), and is therefore a potential therapeutic agent in the treatment of depression. These properties position fucoidan as a promising candidate for MDD intervention, though clinical trials are needed to validate these preclinical findings.

### 1.6. The Effects of Sulforaphane on the Blood-Brain Barrier

Sulforaphane, a bioactive isothiocyanate found in cruciferous vegetables, has shown great promise in protecting the BBB and improving cognitive function in several models of brain injury. Sulforaphane activates the nuclear factor erythroid 2-related factor 2 (Nrf2) pathway, leading to increased expression of cytoprotective genes and antioxidant enzymes [77], bolstering cellular resistance to oxidative insults. This activation helps maintain the integrity of the BBB, reduces cerebral edema, and protects against stroke-induced damage [68]. Moreover, it has been demonstrated that Nrf2 activation with sulforaphane, in vivo and in vitro, upregulates the expression and transport activity of three ATP-dependent drug efflux pumps at the BBB: P-glycoprotein (Abcb1), [Mrp2 (Abcc2)], and [Bcrp (Abcg2)] at blood–CNS barriers [78].

This selective upregulation of efflux transporters in response to oxidative stress may enhance neuroprotection by strengthening the barriers, leading to a 50% reduction in drug accumulation in the CNS and a 150% increase in transporter activity.

Moreover, sulforaphane was found to selectively inhibit MMP-9 secretion in brain endothelial cells (HBMECs) [79]. This is of high importance considering that MMP-9 plays a pivotal role in BBB disruption by degrading TJ proteins and the basement membrane, leading to increased permeability and allowing harmful substances to enter the brain [80]. This mechanism may contribute to the promotion of neuroinflammation and is implicated in various clinical conditions associated with elevated systemic inflammation. Additionally, it was reported that sulforaphane can reduce HBMEC migration, which may enhance BBB protection, modulate neuroinflammation, and provide therapeutic benefits for CNS disorders. Sulforaphane has also shown potential in treating autoimmune conditions, such as experimental autoimmune encephalomyelitis, by reducing oxidative stress, preserving BBB integrity, and modulating inflammatory responses [81].

In addition to sulforaphane’s direct effects on the BBB, it has been documented that repeated sulforaphane administration exerts antidepressant- and anxiolytic-like effects by inhibiting HPA axis hyperactivity and restraining inflammation in stressed mice, suggesting its neuroprotective properties [82].

Overall, sulforaphane has demonstrated great potential to protect the BBB through the activation of the Nrf2 pathway, enhancing antioxidant defense, and upregulation of efflux transporters (Table 1). Its ability to inhibit MMP-9 secretion and reduce endothelial cell migration supports BBB integrity and neuroinflammation modulation (Figure 2), making it a promising therapeutic candidate for CNS disorders. Nonetheless, further clinical studies are warranted to confirm these preclinical findings and explore optimal dosing strategies, pharmacokinetics, and long-term efficacy in humans.

### 1.7. The Effects of Vitamin D on the Blood-Brain Barrier

A recent review showed that vitamin D deficiency is common among children, adolescents, middle-aged, and older adults globally, with a particularly high percentage observed in subjects with depression [83]. Moreover, this deficiency correlates with elevated inflammatory markers in depressed and suicidal individuals and with depressive symptom severity [84]. This aligns with findings that vitamin D regulates the genetic processes responsible for the synthesis of key neurotransmitters, including acetylcholine, dopamine, serotonin, and gamma-aminobutyric acid (GABA), all of which are crucial for modulating neuropsychological functions such as mood, cognition, learning, memory, reward processing, and sleep [85]. Furthermore, vitamin D supplementation combined with fluoxetine is more effective in alleviating depressive symptoms than fluoxetine alone in patients with depression [86]. This has sparked considerable interest in the potential efficacy of vitamin D in treating depression. Clinically, vitamin D supplementation has been shown to enhance the efficacy of fluoxetine in patients with depression, and high-dose supplementation (100 µg/day) over several months has improved depressive symptoms in multiple trials [87,88].

The direct effect of Vitamin D on the BBB has been documented in several preclinical studies. In a rodent model of traumatic brain injury (TBI), vitamin D3 supplementation reduced BBB disruption by preserving tight junction (TJ) proteins such as occludin and ZO-1 and reducing brain edema and inflammation, including the inhibition of tight junction protein degradation such as ZO-1 and occludin [89]. Additional studies suggest that vitamin D supplementation holds potential for improving neurovascular coupling and BBB function. It has been shown that HBMECs express VDR, implicating vitamin D in the regulation of both BBB transporters (e.g., P-glycoprotein) and TJ proteins (occludin, claudin-5, and ZO-1) [90]. Additionally, vitamin D downregulates adhesion molecules, including ICAM-1 and VCAM-1, in endothelial cells, thereby limiting immune cell migration into the CNS [91]. Therefore, vitamin D could be essential in counteracting the damaging cascade of injury processes and functional impairments resulting from compromised BBB integrity.

Vitamin D plays a significant role in regulating folate transport across the BBB by upregulating the expression of the reduced folate carrier (RFC). Activation of the vitamin D receptor (VDR) by calcitriol, the active metabolite of vitamin D, increased RFC mRNA and protein expression, leading to increased RFC activity in BBB models, including human cerebral microvascular endothelial cells (hCMEC/D3) and mouse brain capillaries [92]. This mechanism may have clinical utility in enhancing brain folate delivery, critical for neurometabolic health, particularly in the context of depression, where folate deficiency is common and associated with poorer antidepressant outcomes. Importantly, folate deficiency, which is more common in depressed patients and linked to poorer antidepressant response, may limit the efficacy of folate supplementation unless vitamin D status is also considered [93]. Further research is needed to determine optimal dosage and long-term implications of folate supplementation, particularly regarding vitamin D’s role in folate transport.

Additional study highlighted the role of 1,25-(OH)2D3 in maintaining BBB integrity, particularly in the context of multiple sclerosis (MS) [94]. Breakdown of the BBB, which subsequently induces inflammation in the CNS, is an important step in MS onset or relapse. The authors found that 1,25-(OH)2D3 effectively restores the expression of TJ proteins, claudin-5 and ZO-1, in endothelial cells exposed to serum from MS patients. Moreover, 1,25-(OH)2D3 directly inhibits the nuclear translocation of NF-κB, a critical mediator of the inflammatory response [94]. This inhibition reduced the expression of VCAM-1 and phosphorylated NF-κB (p-NFκB), thereby preventing BBB disruption in both relapsing-remitting MS (RRMS) and secondary progressive MS (SPMS). These findings support the therapeutic potential of calcitriol in modulating inflammation and preserving BBB function in MS [94]. This positions the active form of vitamin D as a potential therapeutic agent for BBB disruption.

In conclusion, vitamin D3 is crucial for maintaining BBB integrity and shows promise in treating CNS disorders like depression. It regulates TJ proteins, adhesion molecules, and folate transporters (Figure 2), reducing BBB disruption and inflammation (Table 1) in models such as traumatic brain injury and multiple sclerosis. Its ability to modulate neuroinflammation and improve cognition supports its role as an adjunct therapy. However, more clinical and preclinical studies on its direct effects on the BBB are urgently needed.

### 1.8. The Effects of Urolithins on the Blood-Brain Barrier

Urolithins (Uros), gut metabolites derived from ellagitannins (ETs) and ellagic acid (EA), are present in foods like pomegranates, berries, walnuts, and wood-aged wine and in medicinal plants such as Galla chinensis, Chebulae fructus, and seabuckthorn leaf [95]. ETs and EA have limited bioavailability in vivo and are metabolized into Uros by colon microbiota [96]. However, the bioconversion of Uros from ETs and EA varies significantly among individuals and is primarily attributed to individual differences in gut microbiota composition shaped by age, health status, diet, obesity, and gastrointestinal surgeries [97].

In the context of psychiatric disorders, one study investigated the impact of urolithins on monoamine oxidase (MAO) activity, an enzyme whose increased activity contributes to the inactivation of neurotransmitters in disorders such as depression and Parkinson’s disease. This study identified urolithin B as the most effective MAO-A inhibitor. This inhibition may elevate neurotransmitter levels (e.g., Serotonin, dopamine), thereby contributing to mood stabilization and the alleviation of depressive symptoms, highlighting its potential to maintain or increase neurotransmitter levels and, consequently, improve mood [98].

Additional studies have investigated the effects of urolithins on BBB parameters. Specifically, Uros has been found to reduce brain edema and protect BBB function by minimizing the disruption of key TJ proteins, such as occludin and ZO-1 [99]. As mentioned, neuroinflammation plays a pivotal role in the breakdown of the BBB, with NF-κB being one of the key regulators of this inflammatory response [100]. Specifically, the Akt/IKK/NF-κB pathway regulates NF-κB activation, and activated Akt phosphorylates the IKK complex, degrading IκB, which allows NF-κB to translocate into the nucleus and triggers inflammation [101]. A growing body of evidence showed that Uros inhibits Akt activation [102], which further downregulates IKKα and NF-κB phosphorylation. These data indicate that Uros protects BBB function by limiting inflammation through the blockade of the Akt/IKK/NF-κB pathway. Furthermore, it has been found that Urolithin A attenuates BBB leakage induced by TBI by increasing TJ proteins, including occludin and ZO-1 [99].

Overall, urolithin A has been shown to reduce brain edema and protect the BBB by mitigating TJ disruption and inhibiting the Akt/IKK/NFκB signaling pathway (Figure 2), thereby reducing neuroinflammation and enhancing neuronal survival (Table 1). Despite encouraging preclinical results, further clinical validation through well-structured trials is essential to confirm urolithin’s therapeutic potential in MDD.

### 1.9. The Effects of PUFAs on the Blood-Brain Barrier

Omega-3 polyunsaturated fatty acids (n-3 PUFAs) are a group of essential fatty acids that cannot be synthesized by the human body and must therefore be obtained through the diet [103]. Omega-3 fatty acids include docosahexaenoic acid (DHA) and eicosapentaenoic acid (EPA), which are derived from alpha-linolenic acid (ALA) [104].

Over the past decades, n-3 PUFAs have gained attention as promising agents in both the prevention and treatment of depression. Numerous epidemiological and clinical studies have highlighted their beneficial effects, coupled with a favorable safety and tolerability profile [105,106]. Despite this encouraging evidence, the exact mechanisms through which n-3 PUFAs exert their antidepressant action are still not fully understood. Current evidence suggests that n-3 PUFAs may influence depression by modulating inflammatory pathways and oxidative stress responses [106], enhancing neuroplasticity [107], and regulating the hypothalamic-pituitary-adrenal (HPA) axis [108]. However, whether part of their efficacy is mediated through stabilization of the blood-brain barrier (BBB) remains an open and compelling question.

In pathological conditions, BBB integrity is lost through a range of mechanisms that include dysregulation of matrix metalloproteinase-9 (MMP9), defective regulation of aquaporin-4 (AQP4), and altered expression of the ATP-binding cassette transporter B1 (*ABCB1*) gene [109]. Specifically, MMP9 promotes disruption of the BBB by breaking down tight junction proteins and basement membranes [80]. This permeability defect allows peripheral immune cells and toxic substances into the brain, initiating glial activation and neuroinflammatory pathways [110]. It was recently demonstrated that n-3 PUFAs can reduce MMP9 synthesis by suppressing transcription factors such as AP-1 and NF-κB, which are attached to the *MMP9* gene promoter [111]. This reduces *MMP9* gene expression. Furthermore, n-3 PUFAs may reduce MMP9 levels through the reduction of proinflammatory cytokines such as IL-1β and IL-6. Therefore, the reduction of MMP9 in the extracellular space inhibits its proteolytic activity at the BBB, preserving its structural integrity and alleviating depressive symptoms (Figure 2).

Apart from their effect on MMP9, n-3 PUFAs also affect other key regulators of BBB function.

For instance, they control AQP4, a water channel critical for glymphatic clearance and brain homeostasis. n-3 PUFAs help in maintaining AQP4 expression and polarity, allowing effective clearance of neurotoxic waste products such as amyloid-beta (Aβ) and maintaining astrocyte health [112,113]. By restoring AQP4 localization and dynamics of water transport, n-3 PUFAs might protect cognitive function and reduce inflammation-induced brain injury [114].

Regarding drug transport across the BBB, n-3 PUFAs also affect ABCB1 (P-glycoprotein), a significant efflux transporter. Although limited data are available, some research suggests that n-3 PUFAs suppress *ABCB1* gene expression and lower P-gp activity, increasing brain penetration of P-gp substrate antidepressants [115]. Moreover, by integrating into cell membranes and altering lipid bilayer fluidity, n-3 PUFAs could indirectly modulate the localization and function of P-gp [116].

A novel and emerging area of interest concerns the potential regulation of MFSD2A, a critical transporter in the BBB involved in the regulation of transcytosis and the uptake of LPC-conjugated fatty acids, like DHA [117]. As the expression of Mfsd2a prevents BBB disruption by blocking vesicular transport, it is yet to be determined whether n-3 PUFAs regulate Mfsd2a function or expression in depression. This could be another avenue through which n-3 PUFAs stabilize the BBB and evoke antidepressant-like actions. Supporting this hypothesis, a recent publication in Alzheimer’s disease models reported that fish oil (FO) supplementation at high doses caused a significant induction of Mfsd2a in the retina of 5xFAD mice, an AD model animal, compared to controls. This upregulation of Mfsd2a was accompanied by reduced amyloid β (Aβ) deposition in retinal vasculature and normalization of Aqp4, a glymphatic system marker that was elevated in AD models, indicative of glymphatic dysfunction. These findings suggest that n-3 PUFA supplementation through FO can enhance Mfsd2a-mediated regulation of transcytosis, potentially restoring BBB and glymphatic system function, thereby reducing pathological protein accumulation [118]. Although these results were observed in AD models, they provide a promising framework for investigating similar mechanisms in depressive disorders.

Taken together, these findings highlight the multifaceted actions of n-3 PUFAs on BBB integrity via modulation of MMP9, AQP4, ABCB1, and potentially MFSD2A (Table 1). While their use as a standalone treatment for MDD is not currently supported by clinical guidelines, their ability to enhance BBB function and act synergistically with antidepressants underscores their promise as adjunctive therapy. Further studies are warranted to clarify their full therapeutic potential in the treatment of depressive disorders, particularly through the lens of BBB protection and restoration.

### 1.10. Possible Dose-Limiting Side Effects or Suboptimal Efficacy in the Clinical Setting

Despite encouraging results from preclinical studies, translation of nutritional compounds to the clinical setting usually finds issues that can potentially limit their tolerability and efficacy. Some of these issues are less-than-optimal pharmacokinetics, variability in patient responses, and potential dose-related adverse effects. While the selected nutritional components—omega-3 fatty acids, vitamin D, sulforaphane, fucoidan, and urolithins have shown potential neuroprotective and BBB-stabilizing properties in preclinical models, it is useful to note their limitations. Although generally reported as well tolerated, there are findings from studies demonstrating dose-dependent side effects, lack of bioavailability, or mixed results in clinical disease. For example, high-dose vitamin D was associated with an increased risk of falls in elderly populations [119], and the clinical efficacy of omega-3 supplementation in depression remains conflicting between studies [120]. Similarly, sulforaphane also has poor and unreliable bioavailability [121], and the clinical relevance of fucoidan and urolithins is yet to be established [122,123]. Given these considerations, further well-designed studies are required to determine the most effective and safe dosing regimens in clinical settings.

Adding another layer of complexity to clinical translation, epidemiological findings inevitably report a female preponderance of MDD with implications for sex-dependent modulation of BBB integrity. Estrogen has been described to support BBB stability through the maintenance of tight junction protein expression and inhibition of neuroinflammatory responses. However, under conditions of chronic stress, hormonal changes can compromise this protective function, and the barrier dysfunction and increased vulnerability to depression ensue [43,124,125]. Additionally, variability in stress resilience also appears to affect BBB function; resilient individuals have enhanced anti-inflammatory signaling and vascular repair mechanisms, which have potential protective effects against developing depressive pathology [11]. Nutritional interventions rich in omega-3 fatty acids, vitamin D, sulforaphane, fucoidan, and urolithins have the potential to support these protective mechanisms by reducing oxidative stress and inflammation, thereby reinforcing BBB integrity [126]. Nevertheless, the sex-specific effects of such nutritional compounds on BBB function remain poorly understood and warrant further investigation.

Collectively, these findings accentuate the importance of considering biological sex, stress resilience, and compound-specific limitations in creating nutritional or pharmacologic therapies for stabilizing the BBB and treating depression. Increased understanding of these factors will be essential for advancing precision psychiatry and optimizing treatment effectiveness in numerous patient populations.

## 2. Conclusions and Future Directions

Growing evidence underscores the central role of BBB dysfunction in the pathophysiology of MDD, acting both as a consequence and a driver of neuroinflammation and disrupted brain homeostasis. Yet, despite its significance, the BBB remains insufficiently addressed in current therapeutic paradigms.

Several nutraceuticals, including omega-3 fatty acids, vitamin D, sulforaphane, fucoidan, and urolithins, have emerged as promising modulators of BBB function. Their actions include attenuation of inflammatory cascades, reinforcement of tight junction architecture, and normalization of transporter activity. These mechanistic properties provide a compelling rationale for considering dietary interventions as adjunctive strategies in MDD treatment. Although most current evidence stems from preclinical studies, future research must focus on validating these findings in clinical populations, identifying reliable BBB-related biomarkers, and exploring potential synergistic effects with conventional antidepressants. Notably, the ability of these compounds to enhance BBB resilience in sex-specific or stress-sensitive contexts presents a valuable opportunity for the development of targeted therapies. By acting on key pathological processes, such as inflammation, oxidative stress, and barrier disruption, nutraceuticals may help boost antidepressant efficacy, particularly in treatment-resistant cases, minimize adverse effects of conventional antidepressants, and promote neurovascular health. However, the lack of standardized clinical protocols regarding dosing, timing, and drug interactions underscores the need for rigorous translational research to ensure safe and evidence-based integration into routine psychiatric care. Furthermore, nutritional status and eating disorders, especially in pediatric populations, can profoundly affect brain development and quality of life. Recent evidence also highlights the connection between eating disorders and neurodevelopmental conditions such as autism spectrum disorder, which frequently co-occur with emotional and cognitive impairments [127]. This underscores the importance of considering comprehensive nutritional assessments and interventions in the management of neuropsychiatric disorders.

## Figures and Tables

**Table 1:** Summary of key blood–brain barrier (BBB)-related effects of selected nutritional compounds. The table presents the major mechanisms by which each compound influences BBB function, including effects on tight junction (TJ) proteins, inflammatory signaling, oxidative stress, and transporter systems. Data are primarily from in vitro and in vivo preclinical studies. Abbreviations: ↑ increased; ↓ decreased; TJ—tight junction; ZO-1—zonula occludens-1; P-gp—P-glycoprotein; ROS—reactive oxygen species; Mrp2—multidrug resistance-associated protein-2; Bcrp—breast cancer resistance protein; Nrf2—nuclear factor erythroid 2-related factor 2; ICAM-1—intercellular adhesion molecule 1; VCAM-1—vascular cell adhesion molecule 1; MMP-9—matrix metalloproteinase-9; AQP4—aquaporin-4.

Compound/ Mechanisms of Action	TJ Protein Modulation	Inflammatory Pathways	Oxidative Stress	BBB Transporters	MMP-9/AQP4
Fucoidan	↑ ZO-1	↓ neuroinflammation	↓ ROS,	Limited or no data	Limited or no data
Sulforaphane	↑ TJ protein expression via Nrf2 signaling	↓ NF-κB, ↓ neuroinflammation	Potent Nrf2 activator, ↓ oxidative stress	↑ P-glycoprotein, ↑ Bcrp, and ↑ Mrp2	↓ MMP-9
Vitamin D	↑ ZO-1, ↑ Occludin ↑ claudin-5	↓ NF-κB ↓ neuroinflammation ↓ ICAM-1 and ↓ VCAM-1	↓ ROS,	↑ P-gp expression	Limited or no data
Urolithins	↑ TJ, ↑ ZO-1	↓ NF-κB ↓ neuroinflammation	Limited or no data	Limited or no data	Limited or no data
Omega-3 fatty acids	Limited or no data	↓ neuroinflammation ↓ NF-κB	↓ ROS,	May restore transporter function ↓ P-glycoprotein	↓ MMP-9 ↑ AQP4
